# Fungal Diversity Analysis of Grape Musts from Central Valley-Chile and Characterization of Potential New Starter Cultures

**DOI:** 10.3390/microorganisms8060956

**Published:** 2020-06-24

**Authors:** Dinka Mandakovic, Rodrigo Pulgar, Jonathan Maldonado, Wladimir Mardones, Mauricio González, Francisco A. Cubillos, Verónica Cambiazo

**Affiliations:** 1 Laboratorio de Bioinformática y Expresión Génica, Instituto de Nutrición y Tecnología de los Alimentos (INTA), Universidad de Chile, Santiago 7810000, Chile; dinka.mandakovic@inta.uchile.cl (D.M.); rpulgar@inta.uchile.cl (R.P.); jomaldon@gmail.com (J.M.); mgonzale@inta.uchile.cl (M.G.); 2 FONDAP Center for Genome Regulation, Santiago 7810000, Chile; 3 Diagnofast SPA, Santiago 7810000, Chile; 4 Millennium Institute for Integrative Biology (iBio), Santiago 7500565, Chile; wladimirmardones2@gmail.com; 5 Departamento de Biología, Facultad de Química y Biología, Universidad de Santiago de Chile, Santiago 9160000, Chile

**Keywords:** grape must, high-throughput sequencing, non-conventional yeasts, Sauvignon blanc, *Metschnikowia pulcherrima*, starter culture fermentation, culture-dependent method

## Abstract

Autochthonous microorganisms are an important source of the distinctive metabolites that influence the chemical profile of wine. However, little is known about the diversity of fungal communities associated with grape musts, even though they are the source of local yeast strains with potential capacities to become starters during fermentation. By using internal transcribed spacer (ITS) amplicon sequencing, we identified the taxonomic structure of the yeast community in unfermented and fermented musts of a typical *Vitis vinifera* L. var. Sauvignon blanc from the Central Valley of Chile throughout two consecutive seasons of production. Unsurprisingly, *Saccharomyces* represented the most abundant fungal genus in unfermented and fermented musts, mainly due to the contribution of *S. uvarum* (42.7%) and *S. cerevisiae* (80%). Unfermented musts were highly variable between seasons and showed higher values of fungal diversity than fermented musts. Since microbial physiological characterization is primarily achieved in culture, we isolated nine species belonging to six genera of fungi from the unfermented must samples. All isolates were characterized for their potential capacities to be used as new starters in wine. Remarkably, only *Metschnikowia pulcherrima* could co-exist with a commercial *Saccharomyces cerevisiae* strain under fermentative conditions, representing a feasible candidate strain for wine production.

## 1. Introduction

During alcoholic fermentation of grape musts, sugars are transformed into ethanol and carbon dioxide by the actions of fermentative yeasts, mainly *Saccharomyces cerevisiae*. Wine fermentation is typically carried out by selected *S. cerevisiae* strains as microbial starters. Nevertheless, wine fermentation can be a spontaneous process, when it is carried out by the sequential action of different indigenous yeasts (including *Saccharomyces* or non-conventional yeasts), which are present in the must, and often in greater numbers than *S. cerevisiae*. In fact, non-conventional yeasts contribute to the first stages of fermentation and to the organoleptic characteristics of final wine [1]; however, the high efficiency of *S. cerevisiae* to convert sugars into ethanol and its ability to withstand the adverse stress conditions of fermentation results in the progressive death of other yeast species [2,3,4]. Although *S. cerevisiae* is the primary microorganism responsible to complete wine fermentation, at present, a re-evaluation of the role of non-conventional yeasts in winemaking and their use as selected starters in mixed fermentations with *S. cerevisiae* is being carried out [5]. In this context, non-conventional yeasts have the potential to positively contribute to the wine sensory profile through their distinctive production of secondary metabolites and fine-tune the ethanol and glycerol concentrations in wine [5,6].

In recent years, the development of high-throughput sequencing (HTS) provided a useful tool to describe bacterial and fungal communities present in berries, musts and in different stages of fermentation [7,8,9,10]. In particular, non-conventional yeasts have been characterized as a highly diverse population of microorganisms that can be influenced by numerous factors like grape variety, geographical area, climatic factors, sanitary status of grape berries and agronomic practices, among others [8,11,12,13]. Interestingly, the different yeast communities seem to be stably maintained at distinct viticulture regions; hence, the resulting biodiversity of grapevine-associated microbiota could potentially identify a vineyard, linking the wine characteristics to a terroir [14,15]. Therefore, a detailed taxonomic and functional analysis on the microbiota of grape musts could allow the identification of indigenous yeast strains with the potential to contribute to the regional profile of wines [16,17,18,19].

Even though Chile is currently the seventh largest wine-producing and fifth largest wine-exporting country of mostly medium-quality wines, studies of native microbial ecology in grape musts [20] or during the fermentation process [21] are scarce. To the best of our knowledge, no HTS studies on yeast diversity in combination with a culture-based approach have been conducted in unfermented and fermented musts from Chilean vineyards. In this study, the utilization of cultivation techniques coupled with high throughput internal transcribed spacer (ITS) sequencing allowed us to describe the fungal landscape of the indigenous wine yeasts from the variety of *Vitis vinifera* L var. Sauvignon blanc, the white variety with the largest Chilean wine growing area, with more than 15,000 hectares and 10.7% of the national surface in 2018 [22]. In addition, isolates were characterized in depth to describe their biotechnological potential as new starters in wine.

## 2. Materials and Methods

### 2.1. Sample Collection, Processing, and Physicochemical Analyses

The research site is located on the Central Valley of Chile (Curicó) and was surveyed throughout two consecutive seasons of production (years 2016 and 2017), in dependencies of Viña San Pedro (35°05’S and 71°19’W). Samples were collected from unfermented (M) must of a typical *Vitis vinifera* L. var. Sauvignon blanc and from the fermented wine must at the end of the fermentation using a commercial *Saccharomyces cerevisiae* strain (EF; ~30 days post M). Samples were obtained in triplicate from each fermentation step and were used separately as biological replicates. Sub-samples were used for: (1) community DNA extraction, which were placed into sterile plastic bags and immediately stored in dry ice for transport to the laboratory where they were frozen at - 80 °C until DNA extraction, and (2) for fungal growth, which were placed into sterile plastic bags and immediately stored at 4 °C for transport to the laboratory where they were plated a few days after sampling.

The physicochemical data was obtained from Viña San Pedro domestic analyses that incorporated the Fourier transformed Infrared technology (FTIR) using a wine scan FT 120 Foss. All measurements were validated in agreement to the analyses proposed by the International Wine Organization.

### 2.2. Microbiome DNA Extraction, PCR Amplification, and High-Throughput Sequencing

Microbiome DNA was extracted using DNeasy Blood and Tissue Kit (Qiagen, Hilden, Germany) following manufacturer’s instructions and including mechanic lysis of the samples using disruption spheres (FastPrep-24 MP). Extracted DNA was visualized in Tape Station 2200 (Agilent Technologies, Santa Clara, CA, USA) using Genomic DNA Screen Tape, according to the manufacturer’s indications and quantified by fluorescent probes (Qubit Thermo Fisher Scientific, Waltham, MA, USA).

Internal transcribed spacer (ITS) was amplified using the primer set ITS1 (5’-TCCGTAGGTGAACCTGCGG-3’) and ITS2 (5’-GCTGCGTTCTTCATCGATGC-3’), with a barcode in the forward primer. For the amplification, the kit HotStarTaq Plus Master Mix (Qiagen, Hilden, Germany) was used with the following conditions: 94 °C 3 min, 28 cycles of 94 °C 3 s, 53 °C 4 s, and 72 °C 1 min, followed by an elongation phase of 72 °C 5 min. PCR products were examined in agarose gels (2%). Samples were purified using Agencourt AMPure XP (Beckman Coulter, Brea, CA, USA). DNA libraries were constructed following the protocol TruSeq DNA sample preparation (Illumina, San Diego, CA, USA). Sequencing was performed by MrDNA Next Generation Sequencing Service Provider (Shallowater, TX, USA) on Illumina MiSeq platform in an overlapping 2 × 300 bp configuration to obtain a minimum throughput of 20,000 sequences (reads) per sample.

### 2.3. Sequence Analysis and Taxonomical Assignation

The ITS amplicons were processed and analyzed by adapting previously described protocols [23,24]. Briefly, reads were overlapped by pairs and cleaned out of barcodes. Sequences < 150 bp or with ambiguous assignation were discarded. Valid sequences were grouped using USearch (v. 6.1.544) with 4% of divergence in order to remove chimeras and singletons [25,26]. Finally, sequences were filtered with a minimum quality of 30 (q30) with Mothur v. 1.22.2 [27]. Taxonomical assignation was done using the software Quantitative Insights Into Microbial Ecology, QIIME v. 1.9.1 [28]. Operational Taxonomical Units (OTUs) were identified at 95% identity against Unite Community ITS database (v. 7.2) [29] with USearch v. 6.1.544 [25,26] using default parameters in QIIME. The OTUs with mitochondrial or chloroplast assignation were removed. The OTUs identification numbers, abundance and taxonomy retrieved from UNITE database for all samples are specified in Supplementary Table S1. All ITS sequence data used in this study were deposited in the Sequence Read Archive (SRA) of the National Center for Biotechnology Information (NCBI) under the BioProject accession number PRJNA601147.

### 2.4. Alpha Diversity of Samples

To perform alpha-diversity analyses, each sample was randomly subsampled (without replacement) using the alpha_rarefaction.py script found in QIIME 100 to generate Shannon and Chao1 indices along with the observed number of OTUs at different sampling depths. Rarefaction curves for each of these metrics were obtained by serial subsampling (in increments of 5509 sequences and 10 iterations per increment) to a standardized depth of 55,000 sequences per sample.

### 2.5. Principal Components (PCA)

A Principal Components Analysis (PCA) was performed using R package vegan v. 2.5.6 [30] over R v. 3.6.0 to examine compositional changes in the fungal community between wine unfermented must samples from 2016 and 2017 seasons. Our data consisted of 182 OTUs by a 12 sample matrix.

### 2.6. Co-Occurrence Networks Based on Co-Presence and Mutual Exclusion

To examine changes in the composition of the fungal community, we generated co-occurrence networks as described in Mandakovic et al., 2018 [31]. Briefly, significant co-presences or mutual exclusions across the samples were identified by the CoNet method [32] using a multiple ensemble correlation. Four similarity measures were calculated: Bray Curtis and Kullback-Leibler non-parametric dissimilarity indices; Pearson and Spearman rank correlations. In order to cope with the requirements of the CoNet method [32], a distribution of all pairwise scores between OTUs was computed for all M and EF samples (*n* = 12) and OTUs that occurred in less than eight samples and with relative abundances below 0.01% were discarded. For each edge, 1000 renormalized permutations and bootstrap scores were generated according to [33]. Cytoscape [34] was used for the graphic representation of the networks. OTUs were included as nodes in the networks.

### 2.7. Yeast Isolation and Molecular Identification

To obtain yeast isolates, 100 µL of unfermented must samples from years 2016 and 2017 were diluted 1/10, 1/100, and 1/1000 in a sterile physiological solution and plated in triplicates in WL Nutrient Agar medium supplemented with chloramphenicol (100 mg/L) for yeasts over bacteria growth selection. All plates were incubated at 25 °C for 10 days. Thirty-three colonies with different morphologies were selected and isolated from the plates. Pure isolates were cultured in YPD medium (yeast extract 10 g/L, peptone 20 g/L, dextrose 20 g/L) at 25 °C and stored at -80 °C after addition of glycerol (30% *v/v*). To extract yeast DNA, isolated colonies were resuspended in sterile PBS and boiled for 15 min at 95 °C. Samples were centrifuged at 13,000 rpm for 5 min and the supernatant was used as DNA template. For ITS PCR amplifications, primers ITS-1 (5′-TCCGTAGGTGAACCTGCGG-3′) and ITS-4 (5′-TCCTCCGCTTATTGATATGC-3′) were used. ITS PCR amplifications were carried out in 25 μL volumes containing 200 ng (~ 2 μL) of fungal DNA, 12.5 μL of MixGoTaq Green (Promega, Madison, WI, USA), 8.5 μL of nuclease-free water, and 1 μL of each primer (10 mM). The PCR amplification was performed in MJ Research, Inc. Thermal cycling controller with the following protocol: 5 min at 95 °C, 35 cycles of 95 °C for 30 s, 60 °C for 30 s, and 72 °C for 60 s, and a final extension at 72 °C for 10 min. PCR products were visualized in 2% (*w/v*) agarose gel electrophoresis in Tris-acetate-EDTA (TAE) buffer (1×) and stained with ethidium bromide. PCR products were purified and sequenced in Macrogen Korea, while the identification of genus and species was based on the best sequence match obtained using NCBI database. Isolate sequences obtained in this study are available in NCBI database (see Supplementary Table S5). In addition, the reads from the ITS1/2 amplicon sequencing of yeast population were mapped to the ITS1/4 sequences of isolates using BLASTN.

### 2.8. Microcultivation Assay

The microcultivation assay was performed as previously described [35]. Briefly, strains were precultivated in 200 μL of YNB medium (0.67% yeast nitrogen base, 2% glucose) for 48 h at 25 °C. Subsequently, strains were inoculated to an optical density (OD) of 0.03–0.1 (wavelength 620 nm) in 200 μL of media (either YNB or synthetic wine must, SWM) and incubated without agitation at 25 °C in a TECAN Sunrise absorbance microplate reader. OD was measured every 30 min using a 620 nm filter. Each experiment was performed in triplicates. Maximum growth rate, lag time, and OD max for each strain were calculated using GrowthRates software with default parameters [36]. Relative growth was estimated compared to YNB (yeast nitrogen base + glucose 2%) and a z-score regression was performed to normalize values within each condition. In all cases, *p* values were obtained using a one-way ANOVA.

### 2.9. Fermentations and HPLC Analysis

Fermentations were carried out as previously described [37]. Briefly, each strain was fermented in quadruplicate in SWM MS300 (Synthetic must contain 300 mgN/L) and prepared according to Rossignol et al [38]. SWM was supplemented with a final concentration of 300 mgN/L of assimilable nitrogen (YAN) corresponding to 120 mgN/L of ammonium and 180 mgN/L of a mixture of 19 amino acids (612.6 mg/L L-proline, 503.5 mg/L L-glutamine, 503.5 mg/L L-arginine monohydrochloride, 179.3 mg/L L-tryptophan, 145.3 mg/L L-alanine, 120.4 mg/L L-glutamic acid, 78.5 mg/L L-serine, 75.92 mg/L L-threonine, 48.4 mg/L L-leucine, 44.5 mg/L L-aspartic acid, 44.5 mg/L L-valine, 37.9 mg/L L-phenylalanine, 32.7 mg/L L-isoleucine, 50.0 mg/L L-histidine monohydrochloride monohydrate, 31.4 mg/L L-methionine, 18.3 mg/L L-tyrosine, 18.3 mg/L L-glycine, 17.0 mg/L L-lysine monohydrocloride, and 13.1 mg/L L-cysteine). The strains were initially grown under constant agitation in 10 mL of SWM for 48 h at 25 °C. Next, 1 × 10^6^ cells/mL were inoculated into 10 mL of SWM (in 15 mL conical tubes) and incubated at 25 °C. Fermentations were weighed every day to calculate the CO_2_ output until the daily CO_2_ loss represented less than 10% of the accumulated CO_2_ loss. Similarly, fermentations in 50 mL were carried out in 250 mL flasks with constant agitation using a magnetic stirrer at 25 °C and 650 rpm (DLAB Scientific, Beijing, China). Final fermented SWMs, were centrifuged at 9000× *g* for 10 min and the supernatant was collected. 20 µL were injected in a Shimadzu Prominence HPLC equipment (Shimadzu, Tokyo, Japan) using a Bio-Rad HPX–87H column to estimate final concentrations of: glucose, fructose, acetic acid, glycerol, and ethanol [39]. Glycerol and ethanol yields were estimated as previously described [37].

### 2.10. Competition Experiments

Relative fitness in fermentation conditions was estimated using a competition assay. For this, strains were initially grown under constant agitation in 10 mL of SWM for 48 h at 25 °C. Subsequently, 1 × 10^6^ cells/mL or 1 × 10^4^ of EC1118 (*hsp12::GFP- KanMX*, [40] and 1 × 10^6^ cells/mL of the competing strain (the four fast fermenters: *Saccharomyces uvarum Torulaspora delbrueckii*, *M. pulcherrima*, and *Zygotorulaspora florentina*) were inoculated into 125 mL of SWM and kept under constant agitation (200 rpm) at 25 °C during five days. Chilean native *S. cerevisiae* isolates were excluded from competition experiments because their potential in wine fermentation has been previously reported [41]. Relative fitness was estimated at 0, 24, 48, 72, and 96 h post-inoculation quantifying the fraction of G418 resistant colonies versus the total number of colonies on YPD-Agar plate (yeast extract 1%, peptone 2%, glucose 2%, and agar 1.5%).

## 3. Results

### 3.1. Fungal Composition in Unfermented and Fermented Musts from Two Consecutive Seasons of Wine Production

**Figure 1:**
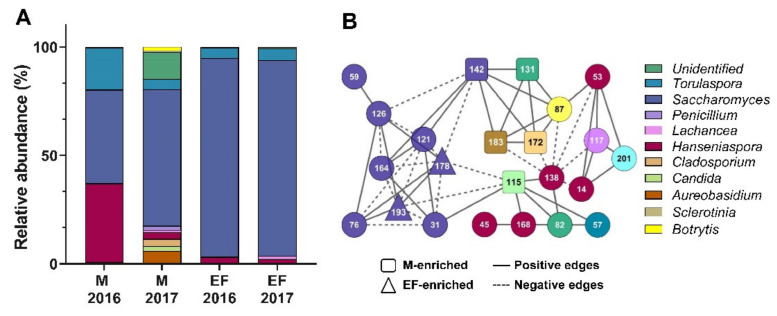
Fungal assemblages of unfermented and end of fermentation stages. (**A**) Average relative abundances of genera from unfermented (M) and end of fermentation (EF) samples (>0.1% of relative abundance in at least one sample) based on massive sequencing of Operational Taxonomical Units (OTUs) in triplicates. (**B**) Fungal interaction network of M and EF samples from both seasons.

We used two types of samples from the wine making process: the unfermented must (M) and the fermented must (wine just before bottling at the end of fermentation using a commercial *Saccharomyces cerevisiae* strain; hereinafter EF). Analysis of the raw sequence data yielded 1,431,165 reads after quality trimming. Taking together both seasons, we identified 182 OTUs (Supplementary Table S1), using a 95% identity threshold against the Unite Community ITS database and with ≥ 0.01% of relative abundance in at least two of the three replicates of M or EF at each season. These OTUs were affiliated to 133 species belonging to 52 genera (Supplementary Table S1) and mainly (>99% of the total relative abundance per sample) to the Ascomycota division. Additionally, microbiome data indicated that 11.5% of the OTUs belonged to unidentified genera (21 out of 182 total OTUs), revealing an important proportion of putative new fungal species in this environment. The most abundant genus in unfermented and fermented musts was *Saccharomyces* (average 53 and 91%, respectively, Figure 1A), mainly due to the contribution of *Saccharomyces uvarum* (42.7%) and *Saccharomyces cerevisiae* (80%).

In the first season, M and EF were both represented by 25 common or core genera (Supplementary Table S1), while 13 were exclusive for M (*Erysiphe*, *Rhodosporidiobolus*, *Kabatiella*, *Trichoderma*, *Talaromyces*, *Leptoxyphium*, *Malassezia*, *Hansfordia*, *Seimatosporium*, *Sarocladium*, *Filobasidium*, *Holtermanniella*, and *Rhodotorula*) and only four very low abundant genera (with less than 0.01% of relative abundance) were exclusive for EF (*Zygoascus*, *Didymosphaeria*, *Cadophora*, and *Curvibasidium*) (Supplementary Table S1). On the other hand, in the second season, M and EF had 23 core genera affiliated, where M had 16 exclusive genera, and EF only had a single low abundant exclusive genus (*Schwanniomyces*, 0.002% of relative abundance) (Supplementary Table S1).

Regarding solely M samples, from the 48 total genera present in this sample, 29 were core between both seasons; meanwhile, nine were exclusive of the first season, and 10 of the second season (Supplementary Table S1). Differences between seasons were also reflected in a significant difference (*p* < 0.05, ANOVA) in richness among the unfermented samples (Supplementary Table S2, Chao-1 index), and in a Principal Component Analysis (PCA; Supplementary Figure S1), in which PC1 explained 80% of the variation in community composition (Supplementary Figure S1). Between seasons, two genera, *Hanseniaspora* and *Torulaspora*, were highly different in their relative abundances among the M samples. Specifically, *Hanseniaspora* accounted for 36.2% of the relative abundance present in year 2016, but only for 3.1% in year 2017, whereas *Torulaspora* accounted for 19.3% of the relative abundance in 2016 and only for 4.8% in year 2017 (Supplementary Table S1, Figure 1A). Concerning the physicochemical parameters of the unfermented musts, they were similar among seasons (Supplementary Table S3).

EF samples showed a different pattern compared to M samples. While 60.6% of the genera from these samples were core (*n* = 20), only nine and four genera were exclusive to the first and second seasons, respectively (Supplementary Table S1). This data was also reflected in the absence of significant difference of richness values between seasons for EF samples (Supplementary Table S2, Chao-1 index). Additionally, in both seasons, the EF Shannon value was significantly lower than in M (*p* < 0.05, ANOVA, Supplementary Table S2), suggesting that a higher diversity was present in unfermented over fermented musts.

The putative ecological interactions among different fungal species within M and EF communities were examined by a microbial interaction network analysis (Figure 1B). For the purpose of having an appropriate number of samples to cope with the requirements of the CoNet method for network analysis [32], the six M and six EF samples from the two consecutive seasons of production were used. Network properties such as the number of links of the node (degree) and interaction type (nodes with positive and negative links, representing co-presences and mutual exclusions, respectively) were listed (Supplementary Table S4). From a total of 182 OTUs identified, 23 OTUs belonging to nine genera were engaged into the network, which showed a higher proportion of positive (*n* = 76) than negative interactions (*n* = 40). It is important to mention that positive or negative interactions within fungal communities can represent ecological interactions (co-presences or mutual exclusions), or simply indicate that they are responding in the same way to a change in environmental conditions [42,43], thus a functional analysis of isolates is needed to interpret these interaction patterns. Interestingly, *Saccharomyces* genus was represented by the greatest number of OTUs (*n* = 9) within the network, exhibiting the highest degree values. Moreover, three OTUs belonging to this genus were enriched (>20 folds) at M or EF or samples (Supplementary Table S4).

### 3.2. Identification of Yeast Species of Unfermented Grape Musts

A total of 33 distinct colonies were isolated from unfermented musts, which were differentiated based on colony color and morphology on WL nutrient agar. The ITS1 and ITS4 regions of the 33 isolated yeasts were then sequenced for species identification. Out of this, we identified individuals belonging to six genera and nine species as follows: *Zygotorulaspora florentina*, *Torulaspora delbrueckii*, *Saccharomyces ovarum*, *Saccharomyces cerevisiae*, *Starmerella bacillaris*, *Metschnikowia pulcherrima*, *Pichia fermentans*, *Pichia membranifaciens*, and *Pichia manshurica.* Supplementary Table S5 provides an overview of the isolated representative strains, their ITS region fragment sizes, and their GenBank accession numbers.

**Figure 2:**
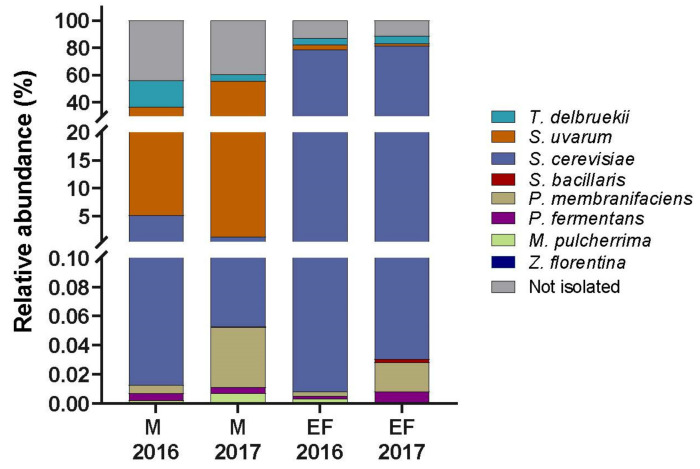
Relative abundance of isolates in unfermented and end of fermentation stages. Reads from high-throughput sequencing (HTS) were mapped to the internal transcribed spacer (ITS) sequences of isolates to calculate the average relative abundances of isolated species from unfermented (M) and end of fermentation (EF) samples. In grey, relative abundance of the remaining species that were not isolated but were massive sequenced.

When reads from HTS were mapped to the ITS sequences of isolates, the relative abundances of the isolated strains in M and EF samples were calculated for years 2016 and 2017 (Figure 2). Eight out of nine isolates were present in M and EF at both sampling seasons, but with different relative abundances. At year 2016, *T. delbrueckii* represented 19.3% and 4.8% of the population in M and EF, respectively, while at year 2017, it was present at similar levels in both samples (4.9% in M and 5.3% in EF). *M. pulcherrima*, *P. fermentans*, *P. membranifaciens*, *Z. florentina*, and *S. bacillaris* were found at very low abundances at both sampling seasons, whereas *S. uvarum* was highly abundant in M samples (31.4% and 54.1% for years 2016 and 2017, respectively) and then decreased in EF samples (3.5% in 2016 and 1.7% in 2017). In contrast, *S. cerevisiae* increased in abundance to become the dominant genus in the EF samples (78.6% in 2016 and 81.5% in 2017). One of the isolates, *P. manshurica*, was the only species gained in culture but absent from the microbiome sequencing. This may be due to a low efficiency of DNA isolation from cells of this species and/or to a very low abundance of this yeast in the unfermented musts samples, which could have prevented its detection by the sequencing strategy.

### 3.3. Fermentation Performance of Natural yYeasts Isolates on Synthetic Wine Must MS300

**Figure 3:**
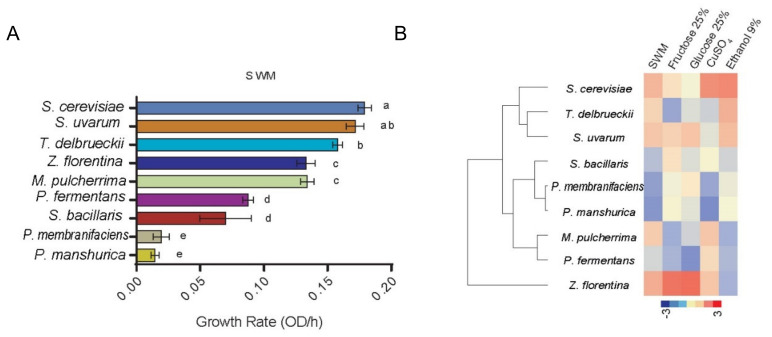
Wine-related phenotypes in natural strains. (**A**) Maximum growth rate (µmax, OD/h) in synthetic wine must (SWM), a,b,c,d,e depict significant differences (p-value < 0.05, ANOVA). (**B**) Heatmap from all phenotypes evaluated under micro-cultivation conditions.

In order to determine the ability of isolated yeast to generate biomass under wine must fermentation and laboratory growth conditions, a high-throughput microcultivation assay was performed under synthetic wine must MS300 (SWM) and YNB media, respectively, and from this we determined: lag phase, maximum growth rate (µmax), and maximum OD (OD max) (Supplementary Table S6). Interestingly, some non-conventional species such as *Torulaspora delbrueckii* showed high µmax and OD max under SWM (*p* value < 0.05, ANOVA) like *Saccharomyce*s strains, suggesting a high degree of adaptation to this hostile environment (Figure 3A). For *Pichia* species a low µmax was observed under SWM, except for *Pichia fermentans* which showed a greater µmax compared to the other three *Pichia* species analyzed. On the contrary, under laboratory conditions (YNB + 2% Glucose), *Pichia* strains showed a higher µmax and OD max relative to SWM (Supplementary Table S6). Subsequently, we evaluated a series of conditions normally found in wine: the hyperosmotic stress response in glucose 25% and fructose 25%, together with ethanol resistance (ethanol 9%) and estimated their relative growth rate (µmax) to YNB supplemented with glucose 2% (Figure 3B). Interestingly, two *Pichia* strains *(P. membranifaciens* and *P. manshurica*) clustered separately from the other strains and were tolerant to hyperosmotic and ethanol 9% stress, where hyperosmotic stress could explain their prevalence in non-fermented wine must (high hyperosmotic media). On the other hand, *T. delbrueckii* clustered close to *Saccharomyces* strains, exhibiting a high relative growth level under most conditions, except for fructose 25% where lower µmax were observed (Supplementary Table S6). *M. pulcherrima* clustered together with *P. fermentans*, mostly due to a low hyperosmotic stress, ethanol tolerance and fructose uptake. *Saccharomyces* strains showed the highest ethanol and SWM µmax, clustering together for all estimated parameters (Supplementary Figure S2). These results demonstrate the dominance of *Saccharomyces* strains across wine-related phenotypes; however, it also highlights that other non-conventional yeasts are able to tolerate high sugar and/or ethanol levels.

**Figure 4:**
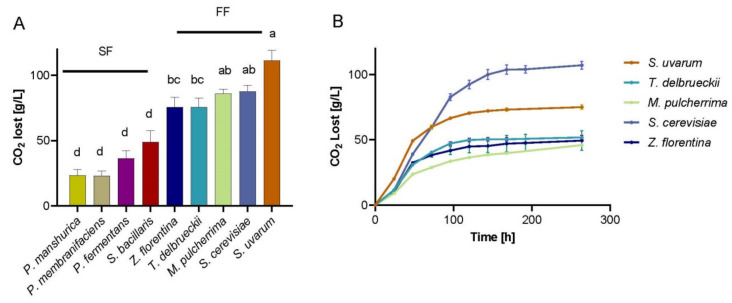
Fermentative capacity of natural strains. (**A**) Total CO_2_ lost in all strains in 10 mL fermentations (SF, Slow Fermenters and FF, Fast Fermenters), a,b,c,d,e depict significant differences (p-value < 0.05, ANOVA). (**B**) FF under 50 mL fermentations with agitation. EC1118 was used as the commercial *S. cerevisiae* control.

Since most strains were able to grow under microcultivation conditions, we next evaluated their fermentation capacity under micro-fermentations in SWM. For this, 1 × 10^6^ cells/mL of each strain were inoculated in 10 mL and micro-fermentations were carried out at 25 °C for ~21 days. Subsequently, we estimated the CO_2_ loss for the fermentation period representing a fermentation efficiency trait. Overall, we distinguished two groups clearly differentiated based on their total CO_2_ release: fast fermenters (FF) and slow fermenters (SF) (Figure 4A). Among the FF species, we observed the two *Saccharomyces* species: *S. cerevisiae* and *S. uvarum* followed by three non-conventional species: *T. delbrueckii*, *M. pulcherrima*, and *Z. florentina*. The SF group contained four non-conventional species: *T. delbrueckii*, *M. pulcherrima*, and *Z. florentina*. The SF group contained four non-*Saccharomyces* species: *S. bacillaris* and the three *Pichia* species, *P. fermentans*, *P. membranifaciens*, and *P. manshurica*. In agreement with the results obtained under microcultivation conditions, the *Pichia* sp. showed almost none or low CO_2_ loss levels (Figure 4A), demonstrating their incapacity to colonize the wine fermentation environment.

To evaluate the whole fermentative potential of each individual under SWM, we measured fermentation performance of the FF species under a greater SWM volume (50 mL) in constant agitation and included the commercial, and widely characterized, *S. cerevisiae* strain (EC1118) as comparison control (the isolated *S. cerevisiae* strain was excluded from our analysis). Interestingly, *S. uvarum* exhibited greater CO_2_ loss levels than any of the other species, except for the renowned commercial wine yeast (Figure 4B), demonstrating its potential as top fermenter. On the other hand, the three non-conventional species showed lower CO_2_ loss levels compared to the *Saccharomyces* species, exhibiting a lower fermentation performance (Figure 4B). These results demonstrate the greater fermentation capacity of native *Saccharomyces* strains compared to other non-conventional isolates. Still, the latest are able to efficiently ferment the SWM and represent new candidate strains for wine fermentation.

### 3.4. Determination of Organic Acid Composition in Synthetic Wine

**Figure 5:**
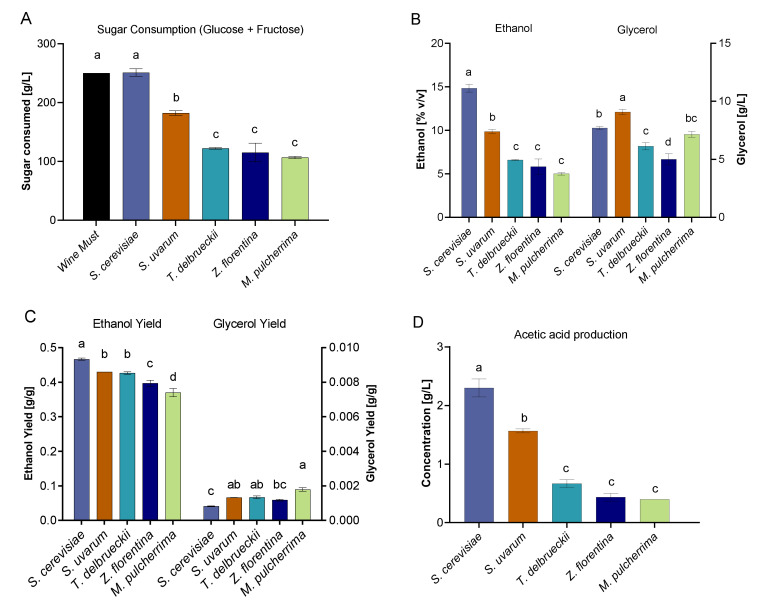
HPLC results from fermentation with *Saccharomyces* and non-*Saccharomyce*s strains. (**A**). Sugar consumption, (**B**) Ethanol and Glycerol production, (**C**) Ethanol and Glycerol yields, and (**D**) Acetic acid production.

Fast fermenters represent potential new starter strains for a controlled fermentation process [3]. In order to determine their ability to consume sugar sources provided in the SWM and the resultant production of other organic acids, we estimated through HPLC the concentrations of important metabolites such as: glucose, fructose, trehalose, acetic acid, ethanol, and glycerol at the end of the fermentation (Supplementary Table S7). As expected, all FF species consumed at least ~50% of the glucose provided, in contrast to fructose where some strains consumed less than 40% of the provided amount (Figure 5A). The commercial isolate consumed all the available sugars (250.9 g/L), while *S. uvarum* consumed only 67% of fructose and glucose.

The production of glycerol and ethanol represent attractive metabolites during wine fermentation, while acetic acid represents an undesirable trait. Interestingly, for *M. pulcherrima* the consumption of glucose and fructose translated into the highest glycerol yields among all strains (*p*-value < 0.05, ANOVA), with over 2.23 times more than the one obtained by EC1118. In contrast, ethanol levels for *M. pulcherrima* were significantly lower compared to *Saccharomyces* species (*p*-value < 0.05, ANOVA, Figure 5B); however, both species exhibited similar yield values (*p*-value > 0.05, ANOVA, Figure 5C), and did not reach commercial standards (Supplementary Table S7). *T. delbrueckii* and *Z. Florentina* also showed greater glycerol levels compared to ethanol, yet in all cases glycerol levels were lower compared to EC1118 and *S. uvarum* (Figure 5B). Interestingly, with both species higher yield values were observed when compared to EC1118 (Figure 5C and Supplementary Table S7). On the contrary, acetic acid levels were significantly high in the two *Saccharomyces* species, while the non-*Saccharomyce*s showed less than 1 g/L of this undesired compound (Figure 5D). In addition, the *S. uvarum* isolate also exhibited high production levels of ethanol and glycerol; however, this strain showed significantly lower levels of acetic acid production compared to the commercial strain under our fermentative conditions. These results demonstrate the potential of the native *S. uvarum* and the non-conventional yeast *M. pulcherrima* for their utilization as starter culture in wine fermentation. However, further assays could be performed in greater volumes under non-synthetic wine must conditions to validate these results.

### 3.5. Competitive Performance of Native Strains in Co-Cultures Systems

**Figure 6:**
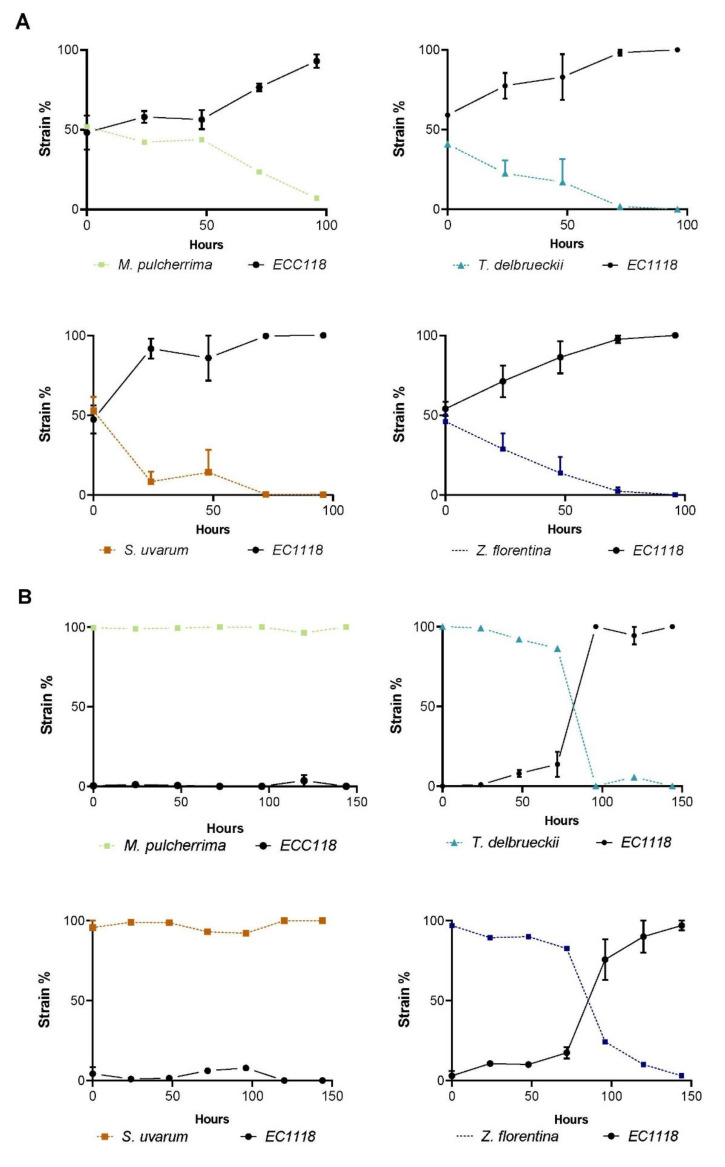
Competition experiment between commercial strains versus natural isolates. The relative percentage of the reporter commercial strain EC1118 (black line) in competition with each of the other FF strains inoculated in (**A**) equal proportions and (**B**) 1:100 proportion.

In order to determine the competitive capacity against *S. cerevisiae* of the non-*Saccharomyces* and *S. uvarum* strains, we co-inoculated in SWM each strain together with a geneticin resistant strain derived from the *S. cerevisiae* EC1118 background [40] and determined their abundance relative to the commercial strain during 100 h. In all cases, the commercial *S. cerevisiae* strain out-competed the others after 48 h, except for *M. pulcherrima*, for which viable cells were found even after 100 h of co-cultivation (Figure 6A). Interestingly, *S. uvarum* was rapidly overtaken by EC1118, representing the strain with the lowest comparative fitness. The *T. delbrueckii* and *Z. florentina* strains showed similar competitive profiles, being overtaken by EC1118 after 72 h. Subsequently, to determine whether the isolated strains were able to dominate the fermentation environment when inoculated in excess compared to EC1118, the co-cultivation assay was modified and 1 × 10^6^ and 1 × 10^4^ cells/mL of non-*Saccharomyces* (and *S. uvarum*) were inoculated together with EC1118, respectively. In this case, *M. pulcherrima* and *S. uvarum* were the sole species able to maintain dominance over the culture and were not dominated by EC1118 (Figure 6B). Altogether, these results suggest that our strain of *M. pulcherrima* represents the sole non-*Saccharomyces* native strain that could co-exist for a longer period in co-cultivation with a commercial *S. cerevisiae* strain under wine fermentative conditions.

## 4. Discussion

In this work, we monitored, during two seasons, M- and EF-associated fungal assemblages from the variety of *Vitis vinifera L va*r. Sauvignon blanc in a commercial winery. Taking into account that the diversity of yeasts during grape must fermentation [21] or associated with grape berries in different Chilean valleys [20] have been described using mainly cultivation techniques and/or qPCR-based assays, here we performed a HTS approach based on the ITS-sequencing of all the eukaryotic microorganisms present in the unfermented and fermented musts in consecutive seasons. Our aims were: 1) to compare M and EF community structures by identifying the core community of indigenous wine yeasts and to determine their putative ecological interactions, and 2) to isolate and characterize cultivable native yeasts as potential new starters for winery industry.

Although only two seasons were analyzed, we observed that M and EF samples shared a common fungal community (core microbiome, 56 OTUs) dominated by species belonging to Ascomycota phylum, while species of Basidiomycota represented less than 0.2% of the population in all samples (Supplementary Table S1), similar to the results that have been reported for different red and white wine grapes [44,45]. Taxonomic analysis also showed that all species of the *Saccharomyces* genus could be recovered consistently in the two sequential seasons in both M and EF samples. The exclusive OTUs found in the M (non-core microbiome) most probably failed to grow up under stress conditions generated during the fermentation process [46].

Additionally, we observed significant seasonal microbial variability in M samples (Supplementary Table S2), such as in genera *Hanseniaspora* and *Torulaspora*, which showed notable differences in relative abundances among seasons (Supplementary Table S1, Figure 1A). This variability could not be explained by the physicochemical parameters measured in these samples, but rather it might be explained by other environmental factors that we did not evaluate and could have affected the microbial community prior to the harvest, such as temperature, relative humidity and rainfall, among others [8,15]. We noticed that from the 48 total genera present in M samples, the exclusive ones from the first and second seasons of the unfermented musts were represented by few reads, adding only 0.004 and 0.012% of relative abundance, respectively (Supplementary Table S1). These results indicate that the differential richness observed among must samples in consecutive years was mainly attributable to rare species that most likely own differential tolerances towards the parameters measured, and thus these factors could have a role controlling the fungal community structure.

Regarding the presence of low abundance taxa, Mendoza et al. (2017) also reported that unfermented samples were characterized by many species represented by very few reads in the ITS sequencing data, whereas yeast communities of fermented samples comprised few species represented by many reads. Accordingly, by the end of fermentation, we observed that the yeast community was practically the same in both years, with no significant differences in Chao-1 nor in Shannon indices (Supplementary Table S2), being *Saccharomyces* consistently the most abundant fungal genus with over 90% of the relative abundance (Supplementary Table S1). Interestingly, nine OTUs belonging to the *Saccharomyces* genus were found, including *S. mikatae* and *S. kudriavzevii*, which may represent the first evidence of the presence of these species in wine-environments in South America [47]. These results demonstrate the potential of the HTS approach to effectively capture the diversity of yeast associated to wine musts and suggest the presence of species which have not been previously isolated in the southern hemisphere. However, the interpretation of these results needs to be taken with caution, especially regarding the presence of *Saccharomyces* species, since low variability has been described for the ITS sequence of species belonging to this genus when compared to other yeasts [48], a feature that might affect species classification. A high abundance of S. *cerevisiae* is expected during the inoculated fermentation, since this fungal species is predominant throughout the fermentation process due to its resistance to elevated amounts of alcohol. In fact, high alcohol contents were measured in both EF samples, with values of 11.4 ± 0.2 and 13.6 ± 1.3% *v/v* for years 2016 and 2017, respectively. In contrast, during the spontaneous fermentation process, *Saccharomyces* dominates later stages of fermentation, whereas non-conventional yeasts can be predominant in the initial and middle stages [49]. These results support the relevance of the fermentation process over yeast selection, and therefore over the structure of the microbial community [14]. Additionally, network analysis enabled us to identify theoretical relationships among the fungal groups [43], showing that members of the *Saccharomyces* genus are forming a cluster or subnetwork module, as expected when species share specific functions with their phylogenetic group [50]. However, the presence of positive and negative links within this module suggests that despite their phylogenetic similarity, they display a differential response (change of their relative abundance) between M and EF states.

Beside *Saccharomyces* species, nine other yeast genera with relative abundances > 0.1% in at least one M and/or EF samples were identified. Among them, species within the genera *Torulaspora*, *Pichia*, *Lachancea*, *Candida/Metschnikowia*, *Aureobasidium*, and *Hanseniaspora* have been described as interesting indigenous yeasts, since they are able to produce desirable compounds and metabolites to improve the wine quality, enzymes with roles in wine production or act as biocontrol agents [12]. In particular, species of *Torulaspora* and *Hanseniaspora*, which can positively influence quality parameters of wine [17,51], were detected in both M and EF samples, but with dissimilar abundances, > 4% and 1%, respectively. In addition to known wine yeasts, we found, mainly in M sample and with low abundance, some other genera such as *Sclerotinia* and *Botrytis* that comprise necrotrophic plant pathogenic fungi with wide host ranges and environmental persistence [52,53] and the genera *Cladosporium* and *Penicillium* encompassing saprophytic molds responsible of organoleptic defects in grapes and wines [54]. All these genera have been previously described as part of the grape and must yeast population [54,55].

In order to select indigenous yeasts to use as potential new starters in the wine industry, we isolated yeasts from the unfermented musts since these samples showed higher richness indices when compared to EF samples. From the nine isolates obtained, only *Pichia manshurica* was not detected by the sequencing strategy. The enrichment of this yeast in the culture medium may be due to the differential supply of nutritional requirements in the culture medium than in the must, the removal of growth inhibitory components and/or the reduction of competitive interactions within members of the community [56]. Additionally, some abundant species of *Saccharomyces* and *Hanseniaspora* genera that were identified in the unfermented musts by the sequencing approach, were not recovered during the isolation process. In this regard, previous reports showed a lack of correlation between the abundance detected by HTS and the possibility of strain isolation [57,58].

As expected, the species of *Saccharomyces* showed high µmax and fermentation rates under SWM. In particular, *S. cerevisiae* strains are extremely efficient during the first 48 h of fermentation because of their fast sugar and nitrogen source uptake [59,60]. These sources are immediately stored in the vacuole and later, during the fermentation process, mobilized out of the vacuole to support growth after depletion of external nitrogen [60]. This ability impedes other yeasts from dominating the culture, making *Saccharomyces* strains best-adapted species to wine must fermentation. Yet, we characterized the fermentation profile and relative fitness of other species found in grape juice that could represent potential new isolates for either controlled or spontaneous fermentations. The fitness of these strains under microcultivation conditions correlated well with their fermentation profiles. *Saccharomyces* strains together with *T. delbrueckii* clustered together in most kinetic parameters estimated (Figure 3B), demonstrating their similar fitness under fermentative conditions.

Three non-conventional yeasts species showed high fermentation rates: *T. delbrueckii*, *M. pulcherrima*, and *Z. florentina*. *T. delbrueckii* isolates are found naturally in many wine-producing areas, and even recently commercial strains have become available, mostly because of their contribution to the aroma profile [16]. Similarly, *M. pulcherrima* (also known as *Candida pulcherrima*) is also available in the market and is well-known for the high esters production, such as the ethyl octanoate (pear-like aroma) [61]. On the other hand, only recently *Z. florentina* has been considered for wine fermentation, exhibiting high glycerol concentrations and low volatile acids [62]. Here, we obtained lower glycerol levels (and yields) and low acetic acid levels compared to other strains, likely due to the low sugar consumption compared to the other strains. However, glycerol yields were above those obtained in the commercial strain. Indeed, the utilization of *Z. florentina* in co-cultures has been suggested as a favorable strategy to mitigate high volatiles acids production by *S. cerevisiae* [63]. In this context, our competition experiment demonstrated that non-conventional strains are overtaken when co-cultivated with a commercial *S. cerevisiae* strain, suggesting that co-cultivation could occur only during the first 2–3 days of fermentation and limiting their utilization (Figure 6). Instead, *M. pulcherrima* demonstrated the greatest fitness compared to the other tested strains when competed against EC1118, being able to survive for a longer period. In this sense, this strain was the sole found to remain after five days in the mixed culture, demonstrating its plausible utilization in co-cultures with *S. cerevisiae* commercial strains. However, further evidence is needed to determine the organoleptic profile and the effect of sequential co-cultures of *M. pulcherrima* with *S. cerevisiae* commercial isolates. Similar studies have demonstrated the co-inoculation potential of these two species, reducing ethanol levels and increased production of acetate esters and higher alcohols in wine [64]. This new data could provide experimental evidence to establish native *M. pulcherrima* as valuable resources for new local wines.

To the best of our knowledge, this is the first time where HTS has been used to describe the microbiota related to wine-environments directly from grape musts in South America. In this way, the combination of a culture-dependent method and HTS approach enabled us to estimate fungal diversity and dynamics in unfermented and fermented musts of a Sauvignon blanc variety from Chile’s Central Valley. Our finding that the indigenous yeast *M. pulcherrima* could co-exist with a commercial *S. cerevisiae* strain provides support for further studies on *M. pulcherrima* contribution to the sensory quality of wine.

## Supplementary Materials

The following are available online at https://www.mdpi.com/2076-2607/8/6/956/s1, Figure S1: PCA ordination diagram of fungal relative abundance data on M samples, Figure S2: Heatmaps for Lag phase and ODmax, Table S1: Taxonomy and average relative abundance of each OTU in M and EF samples from both seasons, Table S2: Alpha diversity. Microbial diversity indicated by Shannon diversity and Chao-1 indices, Table S3: Physicochemical parameters from unfermented musts, Table S4: Network information, Table S5: Identity, ITS region size and GenBank accession numbers of the isolated yeasts, Table S6: Kinetics parameters in all strains obtained from the microcultivation assay, Table S7: Physico-chemical characteristics of SWM for FF strains after 290 h of fermentation.
