# Supplementary material for: Fungal Diversity Analysis of Grape Musts from Central Valley-Chile and Characterization of Potential New Starter Cultures
Source: Microorganisms. 2020 Jun 24;8(6):956. doi: 10.3390/microorganisms8060956 (PMC7356840; doi:10.3390/microorganisms8060956)
Supplement: Supplementary file 1 [file microorganisms-08-00956-s001.zip › Supplementary material_revised/Figure S1.pdf]

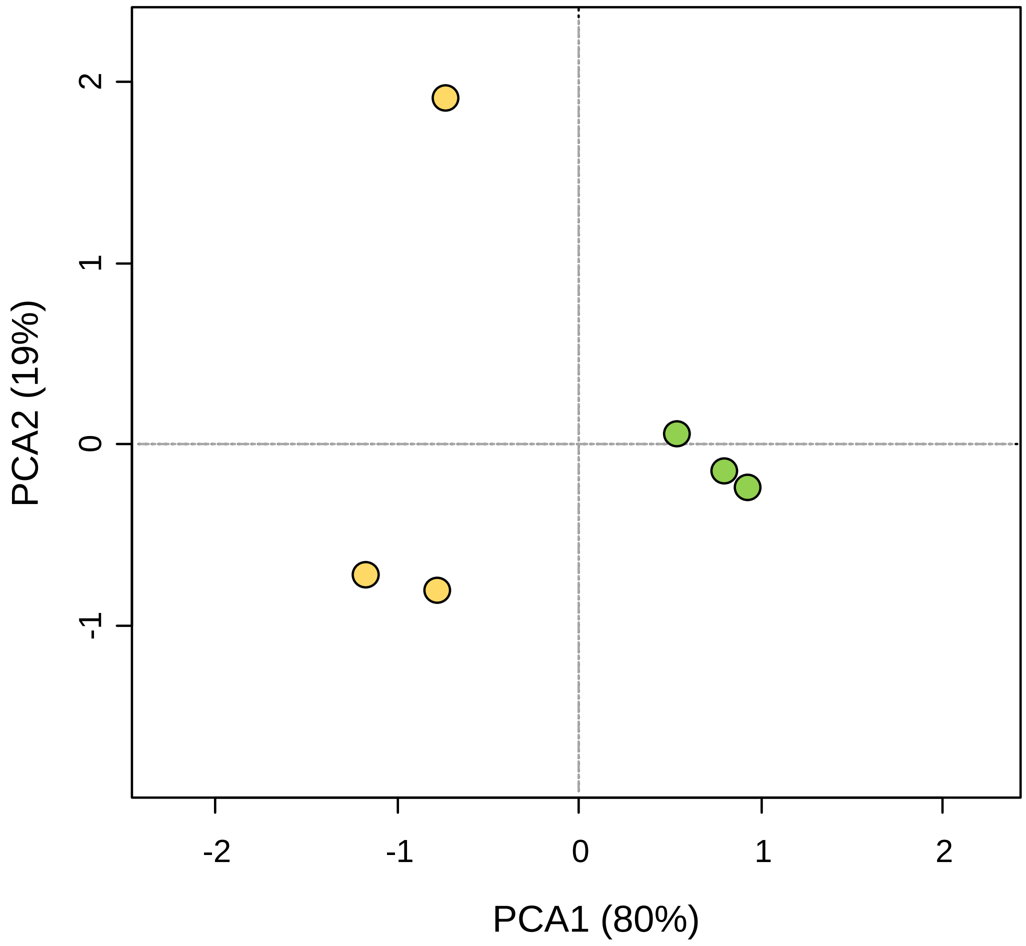

Figure S1. PCA ordination diagram of fungal relative abundance data on M samples (Table S1). Yellow dots represent M samples from the first season; green dots represent M samples from the second season.
