# Supplementary material for: Fungal Diversity Analysis of Grape Musts from Central Valley-Chile and Characterization of Potential New Starter Cultures
Source: Microorganisms. 2020 Jun 24;8(6):956. doi: 10.3390/microorganisms8060956 (PMC7356840; doi:10.3390/microorganisms8060956)
Supplement: Supplementary file 1 [file microorganisms-08-00956-s001.zip › Supplementary material_revised/Table S2.pdf]

**Table S2.** Alpha diversity. Microbial diversity indicated by Shannon diversity and Chao-1 indices. Average values and standard errors are shown. Calculations were based on OTU tables rarefied to the same depth of 55,000 sequences.

| Alpha-diversity index | M 2016 | M 2017 | EF 2016 | EF 2017 |
|---|---|---|---|---|
| Shannon index | 1.32 ± 0.0[a] | 1.42 ± 0.1[a] | 0.72 ± 0.0[b] | 0.60 ± 0.1[b] |
| Chao-1 index | 71.3 ± 7.7[a] | 94.4 ± 2.4[b] | 67.4 ± 10.0[a] | 93.5 ± 11.0[a,b] |

Different letters indicate significant differences between samples for the distinct alpha diversity tests (ANOVA, $p < 0.05$)
