# Supplementary material for: Fungal Diversity Analysis of Grape Musts from Central Valley-Chile and Characterization of Potential New Starter Cultures
Source: Microorganisms. 2020 Jun 24;8(6):956. doi: 10.3390/microorganisms8060956 (PMC7356840; doi:10.3390/microorganisms8060956)
Supplement: Supplementary file 1 [file microorganisms-08-00956-s001.zip › Supplementary material_revised/Table S3.pdf]

Table S3. Physicochemical parameters from unfermented musts.

Physicochemical parameter	Must 2016	Must 2017
Total acidity (g/L)	6.71 ± 0.4	6.43 ± 0.2
pH	3.21 ± 0.1	3.13 ± 0.3
Volatile acidity (g/L)	0.31 ± 0.2	0.29 ± 0.1
Reducing sugars (g/L)	242 ± 9.0	237 ± 6.0
Alcohol (% v/v)	0.00 ± 0.01	0.01 ± 0.01
