# Supplementary material for: Fungal Diversity Analysis of Grape Musts from Central Valley-Chile and Characterization of Potential New Starter Cultures
Source: Microorganisms. 2020 Jun 24;8(6):956. doi: 10.3390/microorganisms8060956 (PMC7356840; doi:10.3390/microorganisms8060956)
Supplement: Supplementary file 1 [file microorganisms-08-00956-s001.zip › Supplementary material_revised/Table S4.pdf]

Table S4. Network information. M and EF enriched correspond to ratios >20 between of the average relative abundance in M and EF of each OUT.

OTU number	Species	Degree	Negative links	Positive links
**183**	***Aureobasidium namibiae***	5	1	4
87	*Botrytis porri*	6	1	5
**115**	***Candida boleticola***	7	2	5
**172**	***Cladosporium ramotenellum***	5	1	4
53	*Hanseniaspora meyeri*	5	1	4
45	*Hanseniaspora osmophila*	1	0	1
168	*Hanseniaspora osmophila*	3	0	3
138	*Hanseniaspora osmophila*	8	6	2
14	*Hanseniaspora uvarum*	4	1	3
117	*Lachancea quebecensis*	4	1	3
201	*Pichia kluyveri*	3	0	3
164	*Saccharomyces bayanus*	6	3	3
**142**	***Saccharomyces bayanus***	8	2	6
126	*Saccharomyces cariocanus*	7	3	4
<u>193</u>	<u>*Saccharomyces cerevisiae*</u>	7	4	3
76	*Saccharomyces cerevisiae*	5	2	3
<u>178</u>	<u>*Saccharomyces cerevisiae*</u>	8	5	3
121	*Saccharomyces eubayanus*	7	4	3
31	*Saccharomyces kudriavzevii*	6	3	3
59	*Saccharomyces mikatae*	1	0	1
57	*Torulaspora delbrueckii*	2	0	2
**131**	**unidentified**	4	0	4
82	unidentified	4	0	4
**Total**			40	76

bold M enriched, underlined EF enriched
