# Supplementary material for: Fungal Diversity Analysis of Grape Musts from Central Valley-Chile and Characterization of Potential New Starter Cultures
Source: Microorganisms. 2020 Jun 24;8(6):956. doi: 10.3390/microorganisms8060956 (PMC7356840; doi:10.3390/microorganisms8060956)
Supplement: Supplementary file 1 [file microorganisms-08-00956-s001.zip › Supplementary material_revised/Table S5.pdf]

Table S5. Identity, ITS region size and GenBank accession numbers of the isolated yeasts.

ID	Species	Identity	Size (bp) ITS region	Accession number
CuricoValley_1	*Zygotorulaspora florentina*	97%	611	MN915116
CuricoValley_2	*Torulaspora delbrueckii*	97%	798	MN915117
CuricoValley_3	*Saccharomyces uvarum*	99%	838	MN915118
CuricoValley_4	*Pichia fermentans*	97%	445	MN915119
CuricoValley_5	*Saccharomyces cerevisiae*	99%	843	MN915120
CuricoValley_6	*Starmerella bacillaris*	95%	460	MN915121
CuricoValley_7	*Metschnikowia pulcherrima*	99%	383	MN915122
CuricoValley_9	*Pichia membranifaciens*	99%	477	MN915124
CuricoValley_10	*Pichia manshurica*	94%	461	MN915125
