# Supplementary figures and images for: Fungal Diversity Analysis of Grape Musts from Central Valley-Chile and Characterization of Potential New Starter Cultures
Source: Microorganisms. 2020 Jun 24;8(6):956. doi: 10.3390/microorganisms8060956 (PMC7356840; doi:10.3390/microorganisms8060956)

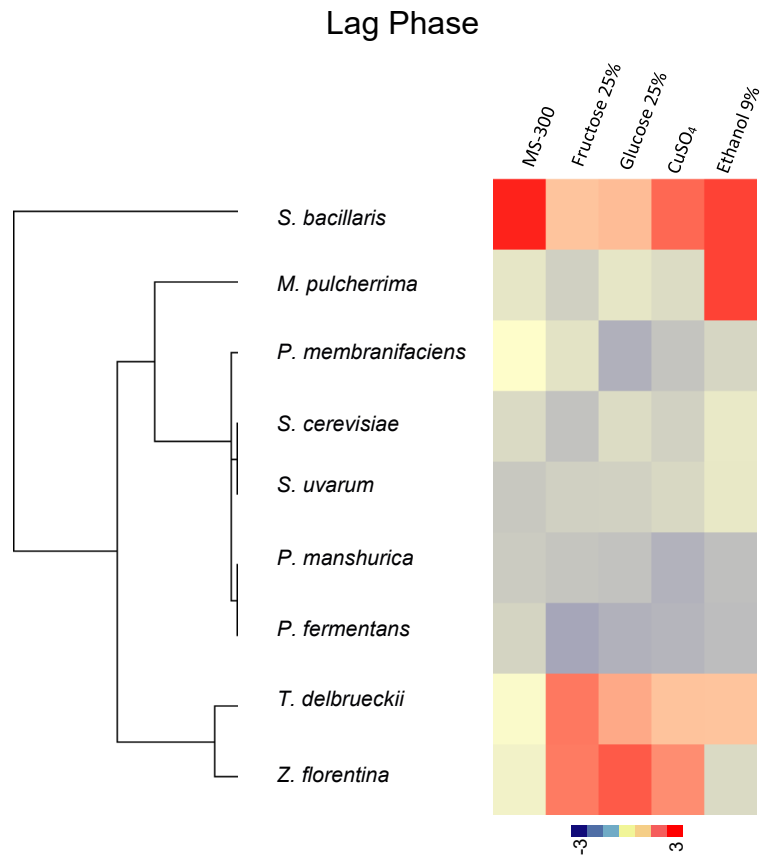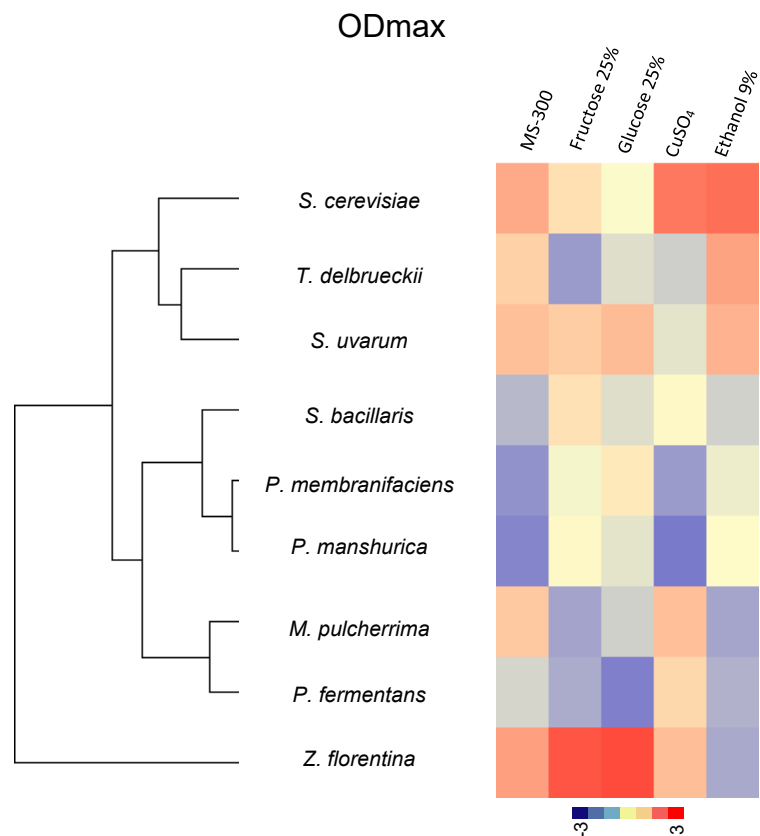

Figure S2. Heatmaps for (A) Lag phase and (B) ODmax.

Supplement: Supplementary file 1 [file microorganisms-08-00956-s001.zip › Supplementary material_revised/Figure S2.pdf]
